# Mobile Delivery of Mindfulness-Based Smoking Cessation Treatment Among Low-Income Adults During the COVID-19 Pandemic: Pilot Randomized Controlled Trial

**DOI:** 10.2196/25926

**Published:** 2021-07-23

**Authors:** Josephine Mhende, Sharrill A Bell, Cherell Cottrell-Daniels, Jackie Luong, Micah Streiff, Mark Dannenfelser, Matthew J Hayat, Claire Adams Spears

**Affiliations:** 1 Georgia State University Atlanta, GA United States; 2 Mindfulness Center of Atlanta Atlanta, GA United States

**Keywords:** acceptability, addiction, African American, cessation, COVID-19, feasibility, income, low socioeconomic status, mHealth, mindfulness, minority, smoking, SMS, text messaging, treatment

## Abstract

**Background:**

Smoking is the leading cause of premature death, and low-income adults experience disproportionate burden from tobacco. Mindfulness interventions show promise for improving smoking cessation. A text messaging program “iQuit Mindfully” was developed to deliver just-in-time support for quitting smoking among low-income adults. A pilot study of iQuit Mindfully was conducted in spring 2020, during the COVID-19 pandemic, among low-income and predominantly African American smokers.

**Objective:**

This pilot study examined the acceptability and feasibility of delivering Mindfulness-Based Addiction Treatment via mHealth during the COVID-19 pandemic.

**Methods:**

Participants were adult cigarette smokers (n=23), of whom 8 (34.8%) were female, 19 (82.6%) were African American, and 18 (78.3%) had an annual income of <US $24,000. They were randomly assigned to either 8 weeks of iQuit Mindfully as a fully automated standalone intervention or iQuit Mindfully in combination with therapist-led in-person group treatment. For participant safety, in-person mindfulness groups were transitioned to the internet and assessments also took place over the internet. Survey questions asked participants about changes in their stress, smoking habits and quit attempts, and their perceptions of the mindfulness and text messaging intervention in the context of the pandemic.

**Results:**

Most participants (n=15 of 21, 71.4%) indicated a change in stress due to the pandemic, of whom 14 (93.3%) indicated higher stress. Participants shared concerns about finances, homelessness, health, and social isolation. Most (n=17 of 21, 80.9%) believed that smoking increases the risk of contracting COVID-19, and although that was motivating for some, others expressed lower motivation to quit smoking because of higher stress. Most (n=18 of 21, 85.7%) stated that practicing mindfulness was helpful during the pandemic. Mean ratings of the helpfulness of text messages and the extent to which they would recommend the program to others were 7.1 (median 8 on a 10-point scale, SD 2.9) and 8.2 (median 9, SD 2.5), respectively. Through open-ended program evaluations, participants shared details about how mindfulness practices and the text messages helped them manage stress and feel a sense of social support during the pandemic. Moreover, 10 of 19 (52.6%) of participants achieved 7-day abstinence from smoking, with no differences between conditions.

**Conclusions:**

This study supports the promise of text messaging and the use of teleconferencing to provide mindfulness and smoking cessation services to underserved populations during a pandemic.

## Introduction

### Background

Smoking continues to be the leading cause of premature death in the United States [1]. In 2015, two-thirds of cigarette smokers were motivated to quit smoking, and over half had attempted to quit in the past year [2]. However, only 7.4% of adult smokers were able to quit successfully [2]. Moreover, low-income adults and members of certain racial and ethnic minority groups (eg, African American individuals) are less likely to quit compared to those with a higher socioeconomic status (SES) and White individuals [2,3]. These priority populations also experience a higher prevalence of tobacco-related illnesses and associated mortality [3]. Barriers to quitting smoking among low-SES populations include high stress as well as low social support and self-efficacy [3-5]. Accessible interventions that directly target these barriers among underserved populations are critically needed, and mindfulness-based approaches might be useful in this regard.

### Mindfulness-Based Interventions for Diverse Populations

Mindfulness is defined as “paying attention in a particular way: on purpose, in the present moment, and nonjudgmentally” [6]. A meta-analysis of randomized controlled trials reported that 25.2% of participants receiving mindfulness interventions for smoking cessation were abstinent 4 months after the intervention as compared to 13.6% of participants who received usual care [7]. Mindfulness interventions have been shown to reduce stress [8], improve social relationship functioning [9], and promote self-efficacy for coping with negative emotions without smoking [10]. Furthermore, mindfulness appears to target addiction by weakening associations of stress and cravings with addictive behavior [11-14]. That is, through mindfulness training, people learn to purposefully respond to stress, cravings, and other unpleasant sensations rather than impulsively reacting by smoking. Mindfulness is also speculated to buffer the negative mental and physical health consequences of stress [15]. This is particularly relevant for marginalized populations who disproportionately experience both acute and chronic stressors [16].

Although the majority of mindfulness studies have included relatively affluent and non-Latino White individuals [17], recent studies support the use of mindfulness-based smoking cessation for socioeconomically and racially or ethnically diverse populations [18-20]. There is still much work to be done to extend the reach and cost-effectiveness of mindfulness interventions. For example, Mindfulness-Based Stress Reduction [21], Mindfulness-Based Cognitive Therapy [22], and Mindfulness-Based Addiction Treatment (MBAT) [20] involve 8 weekly in-person group sessions, each lasting at least 2 hours. mHealth could be useful for increasing access to mindfulness interventions. In particular, text messaging can provide tailored, just-in-time interventions at relatively low cost. Hence, a mindfulness intervention for smoking cessation “iQuit Mindfully” was implemented with strong feasibility and acceptability among low-income, predominantly African American adults [23]. Text messages were developed and iteratively refined on the basis of feedback from the target population [24]. They were designed to be personalized and interactive and could be implemented as a standalone program or as a between-session enhancement to in-person MBAT.

Our team conducted an additional pilot study of iQuit Mindfully, both as a standalone intervention and as an enhancement to MBAT, in spring 2020 to further improve the program. Participants were enrolled in January and February 2020 and began the 8-week treatment intervention on February 13, 2020, in Atlanta, Georgia. During this time, the COVID-19 pandemic began to significantly impact the United States. By mid-March 2020, all 50 states reported confirmed COVID-19 cases [25]. At that time, the governor of Georgia declared a Public Health State of Emergency due to COVID-19 and a few weeks thereafter, a mandatory shelter-in-place order was issued statewide. For participant safety, the in-person mindfulness group sessions transitioned on the internet (via WebEx, although participants chose to join via audio only), and all assessments were conducted on the internet.

### Health Disparities During the COVID-19 Pandemic

Although the pandemic has impacted people worldwide in countless ways, it introduced public health concerns that uniquely affected our participants, who were predominantly African American smokers from low-SES backgrounds. For example, smoking increases the severity of respiratory illnesses, and the World Health Organization summarized the current evidence by stating that “smokers are more likely to develop severe disease with COVID-19, compared to non-smokers” [26]. Furthermore, low-SES and African American populations have experienced disproportionate burden from COVID-19. African American people have contracted COVID-19 at higher rates and had higher rates of COVID-19–related mortality [27]. For example, African American people with COVID-19 in Chicago were 6-fold more likely to die than their White counterparts [28]. The majority (68%) of COVID-19–related deaths in Chicago were of African American individuals, although they comprised only 30% of Chicago’s population [28]. Louisiana reported similar numbers, with African American people representing 70.5% of COVID-19 deaths, although they only accounted for 32.2% of the state’s population [29]. In New York City, the initial epicenter in the United States, The Bronx (which had the highest percentage of racial and ethnic minorities and lowest SES of all 5 of the New York City boroughs) had the highest rates of hospitalization (634 per 100,000 population) and COVID-19 deaths (224 per 100,000 population) [30].

The impact of the COVID-19 pandemic on smoking behavior is still unclear. On one hand, smokers might be more likely to quit owing to concerns of an increased risk of illness with COVID-19. On the other hand, increased stress related to the pandemic could present serious barriers to quitting. For many, the shelter-in-place order meant unemployment, home-schooling, and social isolation. Based on an April 2020 nationally representative survey in the United States, 52% of lower-income adults indicated that they or someone in their household experienced unemployment or a pay reduction because of the outbreak (compared to 32% of those with a higher income) [31]. Only 23% of lower-income adults had an emergency fund to cover illness, loss of employment, or an economic recession, compared to 48% of middle-income adults and 75% of higher-income adults [31]. Women, African American adults, Hispanic adults, those under 65 years of age, and those without a bachelor’s degree were more likely to report financial concerns as a result of the COVID-19 pandemic [31]. Yancy [27] noted that the pandemic will end, but the associated health disparities will continue to be a public health priority.

### The Current Study

In efforts to understand participants’ experiences with the pandemic (and with the iQuit Mindfully intervention during this time), we added measures to assess their experiences specifically during the COVID-19 pandemic. Survey questions asked participants about changes in their smoking habits and quit attempts as well as their perceptions of the mindfulness and text messaging intervention in the context of the pandemic. Given that mindfulness training has been shown to promote more adaptive responses to stress, and that treatment could be offered through mobile technology during shelter-in-place orders, iQuit Mindfully was expected to be acceptable and feasible during the COVID-19 pandemic. Although this was not the original purpose of the study, the timing of our study and COVID-19–specific assessments provide insights into the experiences of low-income, racial and ethnic minority smokers during this time and could inform future intervention efforts.

## Methods

### Participants

This study aimed to recruit a racially and ethnically diverse sample of predominantly low-income adult cigarette smokers who were interested in quitting smoking and lived in Greater Atlanta, Georgia. Inclusion criteria were the following: ages 18-65 years; able to speak, read and write in English; smoking at least 5 cigarettes per day; expired carbon monoxide >6 ppm; motivated to quit smoking within 30 days; and at least sixth-grade health literacy (Rapid Estimate of Adult Literacy in Medicine) [32]. Exclusion criteria were as follows: contraindication for nicotine patches, which were provided to them during the study; problematic substance use (Severity of Dependence Scale score >4) [33,34] or a positive response on at least 2 of the 5 Patient Health Questionnaire Alcohol Abuse/Dependence Scale items [35]; clinically significant depressive symptoms (a 2-item Patient Health Questionnaire score of >3 [36,37]; self-reported diagnosis of schizophrenia or bipolar disorder or the use of antipsychotic medications; and pregnancy or lactation. Individuals currently using tobacco cessation medications and regular (at least weekly) users of tobacco products other than cigarettes were also excluded, although participants were not excluded for the use of e-cigarettes. Individuals did not have to own a mobile phone to participate; they were provided the choice of using their own mobile phone or the one provided to them during the study. This study was approved by the institutional review board of Georgia State University (H19243), and all participants provided written informed consent. This pilot feasibility study was funded by the US National Institutes of Health and is not considered a clinical trial in accordance with the National Institutes of Health’s definition [38].

### Procedures

**Figure 1:**
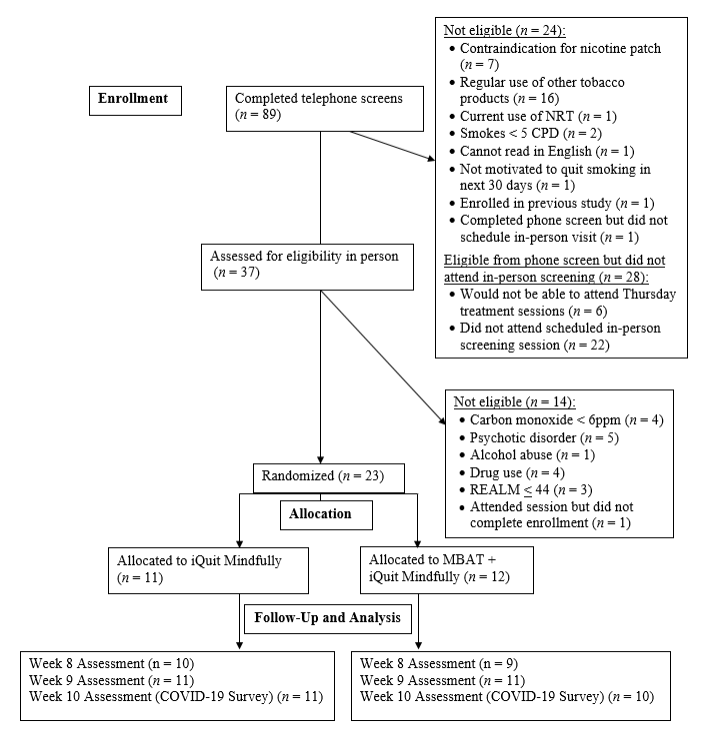
CONSORT flow diagram for recruitment, enrollment, and follow-up assessments. CONSORT: Consolidated Standards of Reporting Trials, CPD: cigarettes per day, MBAT: Mindfulness-Based Addiction Treatment, NRT: nicotine replacement therapy, REALM: Rapid Estimate of Adult Literacy in Medicine.

Recruitment involved the distribution of study flyers in the metro-Atlanta area (eg, downtown Atlanta, near train and bus stops, in the local community health centers) and posted on the internet (eg, Craigslist and neighborhood listservs). Although eligibility was not determined on the basis of income, low-income adults were targeted for recruitment in the study. Interested individuals completed an initial telephone screening, followed by in-person screening (expired CO and assessment of health literacy, mental health, and alcohol or drug use). After informed consent was obtained and baseline assessment was carried out, participants were randomized into 1 of 2 treatment groups (in-person MBAT treatment + iQuit Mindfully text messages [n=12] or iQuit Mindfully alone [n=11]). Figure 1 shows the CONSORT (Consolidated Standards of Reporting Trials) flow diagram. Stratified block randomization was implemented with block sizes of 4 and stratification by race and poverty status. Coauthor MJH generated the random allocation sequence, using SAS software system (version 9.4, SAS Institute). A research staff member (blinded to the size of the blocks) assigned participants to interventions with opaque sealed envelopes marked in accordance with the allocation schedule. Apart from members of the research team who were unmasked to handle randomization and delivery of the interventions, other study personnel were blinded to the treatment conditions. Participants completed in-person assessments at baseline. Remote assessments were carried out on the internet at weeks 8 (end of treatment), 9 (follow-up), and 10 (COVID-19 survey) owing to shelter-in-place restrictions.

### Interventions

All participants received self-help material, nicotine patch therapy, and the iQuit Mindfully text messaging program. Participants assigned to the MBAT + iQuit Mindfully condition also received MBAT treatment for 8 weeks. All participants were asked to set a quit date between 7 and 30 days from the start of treatment. Participants were recruited to begin the interventions all at once (rather than on a rolling basis), and the 8-week treatment began on February 13, 2020.

#### Self-help Material

All participants received the National Cancer Institute’s “Clearing the Air” smoking cessation booklet, including the recommendation to call the Tobacco Cessation Quitline (1-800-QUIT-NOW).

#### Nicotine Patch Therapy

In accordance with the original MBAT protocol [20], all participants were provided nicotine patch therapy for 6 weeks, regardless of treatment condition. Patch therapy for participants who smoked more than 10 cigarettes per day consisted of 21-mg patches for 4 weeks, 14-mg patches for 1 week, and 7-mg patches for 1 week. Patch therapy for participants who smoked 5-10 cigarettes per day consisted of 14-mg patches for 4 weeks and 7-mg patches for 2 weeks. Patch dispensation occurred upon in-person assessment visits. Participants were instructed to apply a new patch each day when they woke up, starting on their quit date, and they were provided detailed paper-based and verbal instructions on the proper use of the nicotine patch. At week 8, 11 of 18 (61%) participants with complete data reported having used nicotine patches in the past week (6 of 9 [67%] of those in the MBAT + iQuit Mindfully intervention and 5 of 9 [56%] in the iQuit Mindfully intervention alone).

#### iQuit Mindfully

iQuit Mindfully text messages [23,24] were sent to all participants each day during the 8-week treatment and 1 week of follow-up. The Upland Mobile Messaging platform was used to generate the automated text message system and send and receive text messages. Text messages were based on the MBAT protocol described below and encouraged participants to practice mindfulness (eg, reminders for informal practice, such as awareness of breath throughout the day, and reminders for formal practice such as the body scan and sitting meditation). They also reminded participants to use specific strategies to aid in cessation (eg, removing cues to smoke, reaching out for social support, and trying other coping techniques from the MBAT protocol [20]). The messages were designed to be interactive; that is, participants were asked questions through a series of flow logic, and they could also text the keywords “CRAVE,” “STRESS,” “SLIP,” or “FACT” at any point to receive an immediate response. Participants could also text keywords (including “MIND,” “BODY,” and “3MIN”) to receive a phone call with a short recording of a mindfulness practice.

Messages were personalized on the basis of first names, personal reasons for quitting, and the amount of money to be saved based on individual smoking habits and price paid per pack. Based on feedback from our previous message testing, participants were able to choose the timing and frequency of text messages. Participants chose from several frequency options (ranging from 1-2 to 5-6 per day) as well as a 12-hour time slot of their choice (either 7 AM to 7 PM or 10 AM to 10 PM). Participants were able to change both the frequency and timing at any point throughout the study. Messages were also personalized on the basis of participants’ chosen quit dates. Each week they were asked whether they had smoked; if they had smoked and their quit date had passed, participants were encouraged to set a new quit date, which was then updated in the text messaging platform. After the initial set-up on the Upland Mobile Messaging platform, the text message intervention was fully automated.

#### MBAT

Participants in the MBAT + iQuit Mindfully condition also received 8 weekly 2-hour group sessions, by a certified Mindfulness-Based Stress Reduction instructor and licensed professional counselor. The MBAT protocol closely follows Mindfulness-Based Cognitive Therapy procedures but replaces depression material with information on nicotine dependence and quitting smoking [20]. MBAT emphasizes personal mindfulness practice in several forms, including sitting meditation, body scan meditation, walking meditation, eating meditation, and gentle yoga or stretching. The program teaches present-focused awareness of moment-to-moment experiences and promotes the ability to purposefully respond to thoughts, feelings, and situations rather than automatically reacting by smoking. For example, MBAT enables mindful awareness of stress, craving, and challenging situations so that participants can more skillfully respond to unpleasant sensations. The first 5 weekly sessions were delivered in person. Because of shelter-in-place orders due to COVID-19, sessions 6, 7, and 8 were delivered through the WebEx teleconference platform.

### Measures

The a priori outcomes for this feasibility study were treatment attendance, retention, and participant feedback on the interventions. Because of the onset of the COVID-19 pandemic and our shift to remote intervention and assessment, this study also focuses on participants’ experiences specifically in the context of the COVID-19 pandemic.

#### Program Evaluations

At week 8, participants completed program evaluations to provide their feedback and suggestions for improving the iQuit Mindfully intervention. They were asked the following: “Of all of the text messages that you received as part of this program, how many did you read?” (response options were “None,” “Some,” “Most,” or “All”); “Overall, how helpful were the text messages in getting you to try to quit smoking?” (rated from 1=“Not at all helpful” to 10=“Extremely helpful”); and “On the scale below, please circle the number that best represents whether you would recommend that other people receive the text messages that you received in this program (or similar texts) as a way to help them quit smoking” (rated from 1=“Would not recommend” to 10=“Would definitely recommend”). MBAT participants were similarly asked about the extent to which they would recommend the MBAT group sessions to others.

#### Smoking Abstinence

At weeks 8 and 9, participants were asked, “In the last 7 days, have you smoked even a puff?” Although biochemical confirmation of smoking behavior had been planned for in-person assessments, this was self-reported owing to web-based or telephone surveys. Missing data were not coded as smoking because of the bias often associated with this “missing=smoking” assumption [39].

#### COVID-19 Survey

At week 10, participants completed a survey of their experiences with stress, smoking, mindfulness practice, and iQuit Mindfully text messages during the COVID-19 pandemic. Participants were asked whether (and how) their level of stress had changed because of the pandemic, with an open-ended follow-up question, “Why do you think your stress level changed?” They were also asked whether their motivation to quit smoking had changed, with a follow-up question of “Why do you think your motivation changed?” Similarly, they were asked about changes in their smoking behavior specifically because of the pandemic, and if so, why. They then answered the following question with a “yes” or “no” response: “Do you think that smoking cigarettes increases a person’s chances of getting sick with coronavirus?” The survey also asked whether mindfulness practice was helpful during the pandemic (and if so, how), whether their mindfulness practice had changed since the pandemic, and which mindfulness practices (if any) they had implemented in the past week. Finally, they were asked whether the text messages were helpful during the pandemic (with responses of “yes” or “no”), and, if so, how.

### Data Analysis

Descriptive statistics were used to characterize the study sample as well as indicators of feasibility, acceptability, and experiences during the COVID-19 pandemic. Illustrative participant quotes were selected from open-ended responses on the program evaluations and the COVID-19 survey.

## Results

### Participant Characteristics

As shown in Table 1, participants were 23 adult cigarette smokers with a mean age of 52 (SD 9.3) years. Slightly over one-third (n=8, 34.8%) were female, and the majority (n=19, 82.6%) were African American. Most (n=18, 78.3%) reported an annual household income of <US $24,000, and 12 (52.2%) were living below the federal poverty level. At baseline, participants smoked 20.7 (SD 12.2) cigarettes per day, and 10 (43.5%) reported smoking their first cigarette within 5 minutes of waking.

**Table 1:** Characteristics of the study participant in the 2 intervention groups (N=23).

Characteristics			Overall		MBAT^a^ + iQuit Mindfully (n=12)		iQuit Mindfully (n=11)
Age (years), mean (SD)			52.0 (9.3)		51.3 (12.0)		52.7 (5.4)
Females, n (%)			8 (34.8)		6 (50.0)		2 (18.2)
**Race, n (%)**							
	African American or Black	19 (82.6)		10 (83.3)		9 (81.8)	
	White	3 (13.0)		2 (16.7)		1 (9.1)	
	Other	1 (4.3)		0 (0.0)		1 (9.1)	
Ethnicity (Hispanic or Latino), n (%)			1 (4.3)		0 (0.0)		1 (9.1)
**Annual income (US $), n (%)**							
	0-2400	4 (17.4)		3 (25.0)		1 (9.1)	
	2401-12,000	9 (39.1)		4 (33.3)		5 (45.5)	
	12,001-18,000	3 (13.0)		1 (8.3)		2 (18.2)	
	18,001-24,000	2 (8.7)		0 (0.0)		2 (18.2)	
	24,001-36,000	2 (8.7)		2 (16.7)		0 (0.0)	
	36,001-54,000	3 (13.0)		2 (16.7)		1 (9.1)	
Below the US federal poverty level, n (%)			12 (52.2)		7 (58.3)		5 (45.5)
**Education level, n (%)**							
	Less than a high school degree	5 (21.7)		2 (16.7)		3 (27.3)	
	High school degree or GED^b^	2 (8.7)		1 (8.3)		1 (9.1)	
	Some college or technical school	7 (30.4)		4 (33.3)		3 (27.3)	
	Associate degree	3 (13.0)		1 (8.3)		2 (18.2)	
	Bachelor’s degree	5 (21.7)		4 (33.3)		1 (9.1)	
	Some graduate school	1 (4.3)		0 (0.0)		1 (9.1)	
**Employment status, n (%)**							
	Regular full-time work (≥40 hours/week)	3 (13.0)		3 (25.0)		0 (0.0)	
	Regular part-time work	2 (8.7)		1 (8.3)		1 (9.1)	
	Student	1 (4.3)		0 (0.0)		1 (9.1)	
	Unemployed	6 (26.1)		4 (33.3)		2 (18.2)	
	Retired	4 (17.4)		1 (8.3)		3 (27.3)	
	Unable to work or disabled	6 (26.1)		3 (25.0)		3 (27.3)	
	Other (self-employed)	1 (4.3)		0 (0.0)		1 (9.1)	
Cigarettes smoked per day, mean (SD)			20.6 (12.2)		23.2 (15.5)		17.9 (6.8)
**Past experience with meditation or yoga, n (%)**							
	Yes	11 (47.8)		7 (58.3)		4 (36.4)	
	Missing	1 (4.3)		0 (0.0)		1 (9.1)	

^a^MBAT: Mindfulness-Based Addiction Treatment.

^b^GED: General Educational Development.

### Retention and Treatment Attendance

Assessment completion rates for the week 8, week 9, and week 10 surveys (all conducted remotely) were 82.6% (n=19), 95.6% (n=22), and 91.3% (n=21), respectively. Among those in the MBAT + iQuit Mindfully intervention, on average participants attended 75% of the first 5 in-person sessions. The median number of in-person sessions attended was 4 of 5. Once treatment transitioned to the internet, participants attended 67% of virtual MBAT sessions (median 2 of 3 virtual sessions attended). Although participants were invited to turn on video mode during the live WebEx sessions, all participants joined via audio only. Participants reported benefits to meeting in this format, particularly in terms of continued social support and community practice time.

### Experiences With Stress in the Context of the COVID-19 Pandemic

Most (n=15 of 21, 71.4%) participants indicated a change in stress because of the pandemic, 14 (93.3%) of whom indicated increased stress. Participants stated that heightened stress was related to concerns about finances, housing, health, and social isolation. One participant reported feeling “isolated and worried about the future” [Participant #214, male], and 2 others stated the following:

I became unemployed, uninsured, and homeless. Moving into homelessness with everything shutting down. Needing to find a place to stay.

Participant #215, female

I was very concerned about catching the virus and how it would [affect] me economically.

Participant #220, male

### Smoking Cessation in the Context of the COVID-19 Pandemic

At week 8, 10 of 19 (52.6%) participants reported that they had not smoked for the past 7 days (7 of 10 [70.0%] in the MBAT + iQuit Mindfully group and 3 of 9 [33.3%] in the iQuit Mindfully group). At week 9, 11 of 21 (52%) participants reported past 7-day abstinence (6 of 11 [54.5%] in the MBAT + iQuit Mindfully group and 5 of 10 [50.0%] in the iQuit Mindfully group). The majority of participants (n=17 of 21, 80.9%) indicated that they believed that smoking increases the risk of contracting COVID-19.

When asked about their motivation to quit smoking, 8 of 21 (38.1%) participants reported that their motivation had changed specifically because of the pandemic. Of them, 4 (50.0%) indicated higher motivation and 4 (50.0%) indicated lower motivation to quit smoking. The other 13 respondents indicated that they were still motivated to quit but had not done so because of COVID-19. For those whose motivation increased, reasons included the following: “my risk of catching the virus. Smoking weakens my immune system” [Participant #203, female] and “because I am concerned about my health…. Smoking in a stressful situation will make it worse” [Participant #212, male]. Among those who indicated lower motivation to quit because of the pandemic, reasons included the following: “the stress has caused me to buy cigarettes” [Participant #220, male] and “my focus needs to be directed to other things first” [Participant #215, female].

Similarly, 8 of 21 (38.1%) participants indicated that their smoking behavior had changed because of the pandemic. Of them, 2 (25%) reported they “quit smoking because of the virus outbreak” [Participant #219, female; Participant #210, male], 4 (50%) reported that they smoked less, and 2 (25%) reported that they smoked more. Reasons for quitting or reducing their smoking because of the pandemic included “money reasons and health reasons” [Participant #201, male] and the following:

Looking at the larger picture, there is a blessing. Smoking will make the virus worse. I have a positive mindset that I will overcome any adversity.

Participant #212, male

It played into quitting. Being sick, not being able to socialize, stress, and coronavirus outbreak played into me wanting to quit smoking.

Participant #210, male

It’s helping my immune system stay strong.

Participant #203, female

For those who reported smoking more or the same amount during the pandemic, explanations included “stress” [Participant #218, female; Participant #221, female; Participant #220, male] and the following:

I'm trying to quit because I can't keep going out like this. I don't trust this virus. I can't smoke with my asthma, it is dangerous. But when I was not stressed I [did] good with not smoking.

Participant #221, female

In the past I was able to channel my energy with work. Once the work was gone I was left with no distraction for my efforts to quit smoking.

Participant #220, male

I'm still trying to slow down. With all that's going on it's not a big priority right now.

Participant #214, male

### Experiences With Mindfulness in the Context of the COVID-19 Pandemic

The majority of participants (n=18 of 21, 85.7%) indicated that practicing mindfulness was helpful during the pandemic. Participants shared details about how mindfulness was helpful for them during the pandemic, including “taking a deep breath and thinking about what you want to do” [Participant #204, male] and the following:

It gives me a chance to be with myself. I can get away from what’s going on in this house and focus just on me.

Participant #208, male

At first I did not know how that was going to fit into my everyday life. It has been extremely helpful. I have more time to think about it. The body scan and other mindfulness practices. That stuff is powerful.

Participant #210, male

Takes me to a quiet place where stress is alleviated. I feel calmer. I feel stronger and more peaceful. Even like my blood pressure has gone down.

Participant #203, female

Sometimes when I was having racing thoughts, I would take me a walk and some deep breaths.

Participant #219, female

I've smoked less per day and with more awareness. I’ve been able to better deal with stress than I thought I would have been able to by practicing breathing and meditation.

Participant #215, female

When asked about whether their frequency of mindfulness practice had changed since the pandemic, more than half (n=12 of 21, 57.1%) indicated that they practiced mindfulness more, while 5 (23.8%) and 4 (19.1%) practiced mindfulness less. At the week 10 assessment, 21 of 23 (91.3%) participants indicated having practiced mindfulness in the past week. Of the specific practices, the most commonly used ones were sitting meditation (n=13 of 23, 56.5%) and awareness of breath (n=12 of 23, 52.2%). Among participants in the MBAT + iQuit Mindfully group, the average rating of whether they would recommend MBAT to others was 9.1 (median 10 on a 10-point scale, SD 2.0).

### Experiences With the Text Messaging Intervention in the Context of the COVID-19 Pandemic

Based on the program evaluation, 15 of 19 (78.9%) participants indicated reading most or all the text messages. The average rating of the helpfulness of the text messages for quitting smoking was 7.1 (median 8.0 on a 10-point scale, SD 2.9), and the average rating of the extent to which people would recommend the text messaging program to others was 8.2 (median 9.0 on a 10-point scale, SD 2.5). When they were asked whether the text messages were helpful specifically during the COVID-19 pandemic, 14 of 20 (70.0%) replied with “yes.” Example responses describing how the texts were helpful during this time include the following:

I felt like I wasn't alone. I still felt like you all were here to support me. The quick keyword responses helped a lot because I didn't have to wait for a response.

Participant #203, female

Reminded me not to pick up a cigarette early in the morning. I reach for my cigarette as soon as I wake up so that text was helpful.

Participant #221, female

Yes, I looked forward to them. It gave me something to look forward to. It helped me feel less stressed.

Participant #202, male

It was my inspiration that I could go another day without buying a pack of cigarettes. I have some of the messages.

Participant #219, female

In terms of engagement with the text messages over the course of the study, all participants interacted with the program by either replying to texts, using keywords, or both. Over the course of 9 weeks, the median number of times participants texted the system was 37 (range 10-261). In addition, 14 of 23 (60.9%) participants used at least 1 keyword (eg, “CRAVE,” “STRESS,” “SLIP,” or “FACT”) to interact with the program.

## Discussion

### Principal Findings

Lower-income and African American populations are faced with a disproportionate burden due to the COVID-19 pandemic [40,41]. Moreover, cigarette smoking is more common in low-income communities [3,42] and has been linked with an increased likelihood of contracting severe disease [26,43]. Low-income adults have already had lower health care access before the COVID-19 pandemic, and this is likely exacerbated during the pandemic owing to issues including loss of employment or health insurance; limited access to telemedicine, and other barriers. In this study on low-income, predominantly African American adult smokers, most participants reported heightened stress because of the pandemic. Specific stressors included homelessness, unemployment, financial concerns, isolation, and worry about the virus. Most participants believed that smoking increased their risk of becoming sick with COVID-19. While this increased motivation among some participants to quit smoking, others indicated lower motivation to quit because of heightened stress during the pandemic.

Our results support the acceptability and feasibility of remotely delivered mindfulness training to address stress and smoking during the pandemic. The retention rate of the web-based assessment time points ranged from 82.6% to 95.6%. Engagement with the mindfulness-based text messaging intervention was high. For those who were receiving group-based mindfulness treatment, attendance was slightly lower in web-based sessions than in the in-person sessions (67% vs 75%, respectively). In program evaluations, participants highlighted the benefits of both mindfulness practice and support from the text messaging intervention in their daily lives. Moreover, at least half of the participants in both treatment arms (mindfulness-based text messaging intervention with or without group treatment) reported achieving 7-day smoking abstinence. Overall, this study supports the promise of text messaging and the use of teleconferencing to provide mindfulness and smoking cessation services to underserved populations during the pandemic. These methods could increase access to evidence-based treatment for underserved populations regardless of pandemic circumstances.

### Comparison With Prior Work

Several studies support the utility of both mindfulness and text messaging interventions for smoking cessation before the pandemic [7,44]. Evidence also supports the efficacy of smartphone apps that advocate mindful acceptance for smoking cessation [45]. Moreover, extant research suggests that mHealth smoking cessation interventions are promising among low- and middle-income countries [46,47], bolstering the notion that mHealth is a viable strategy for targeting smokers with lower socioeconomic resources. While our feasibility study is limited by its small sample size, the rates of self-reported smoking cessation were adequately high. Nine weeks after the start of treatment, 5 of 11 (45.5%) iQuit Mindfully participants and 6 of 11 (54.5%) MBAT participants (all of whom received the iQuit Mindfully text messaging intervention) reported complete abstinence from smoking for the past 7 days. Although not directly comparable because of methodological differences, self-reported 7-day abstinence rates for other text messaging smoking cessation programs have ranged from 20% to 33% [48,49]. For reference, approximately 7% of adult smokers in the United States successfully quit smoking in 2015 [2].

As with our study participants, other studies have reported high levels of stress and distress during the COVID-19 pandemic [50,51]. In particular, African American people have reported higher health concerns related to COVID-19 compared to White people [52]. Low-income and African American communities have already been experiencing a higher prevalence of financial strain, racism, discrimination, and numerous other stressors before the pandemic. Preexisting inequities including lower health care access, higher prevalence of chronic illnesses, low-wage jobs with fewer opportunities to work from home, and lower access to telehealth services have increased during the COVID-19 pandemic [53,54]. Accordingly, community leaders in collaboration with other stakeholders are seeking solutions to help address and process trauma and stress for communities of color. For example, researchers in Michigan have implemented mindfulness and other wellness programs to reduce stress and promote resilience in African American communities during the COVID-19 pandemic [55].

Participants’ descriptions of their experiences with mindfulness and text messaging dovetail with findings from qualitative studies conducted before the pandemic. In previous iterations of the iQuit Mindfully program, participants similarly noted that mindfulness helped them cope with stress, and that the text messages provided important reminders and social support [23,24]. In other text messaging programs for smoking cessation, participants have also highlighted a sense of emotional support [56,57]. In addition, several studies support the utility of mindfulness for addressing mental health among low-income and African American communities [58,59]. Such interventions could be especially relevant in the context of heightened stress during the pandemic.

Digital health interventions involving mindfulness are now being developed and evaluated to address mental health challenges during the pandemic. For example, a web-based intervention is being developed to include mindfulness and cognitive-behavioral skills for addressing COVID-19–related stress [60]. Another study aims to implement a web-based version of a breathing and yoga program for health care workers to reduce anxiety, depression, and insomnia as a result of the pandemic [61]. As this research area grows, it will be important to ensure that effective technologies are accessible to low-income and racial or ethnic minority populations.

### Limitations

This pilot study is limited by its small sample size, lack of biochemical confirmation of smoking status, and short follow-up period (1 week after the end of the program or 9 weeks after the start of treatment). Subgroup analyses could not be conducted owing to the small sample size, but future larger studies might examine whether income or other sociodemographic variables moderate the effectiveness of the intervention. In-person expired carbon monoxide assessment was conducted at baseline but could not be conducted once all procedures became remote owing to the implemented social distancing measures. Nonetheless, this is a timely study on strategies to reach underserved populations when participants experienced heightened stress but were not able to access in-person services. This study could help inform clinicians and researchers working under similar circumstances.

### Conclusions

This study supports the feasibility of a remotely delivered mindfulness intervention to address stress and smoking among low-income adults during the COVID-19 pandemic. This study adds to the relatively small but growing body of research supporting the utility of mindfulness training among low-income and racial or ethnic minority populations. Moreover, mHealth tools could greatly enhance the accessibility of mindfulness interventions for diverse populations. Well-established mindfulness programs typically involve substantial costs and resources (eg, 8 weekly 2-2.5–hour in-person sessions). Text messaging appears to be a low-cost way to provide in-the-moment support to promote well-being in high-stress contexts. This approach could increase treatment access among populations at a higher risk of experiencing adverse effects of the COVID-19 pandemic and other difficult contexts.

## Appendix

Multimedia Appendix 1 CONSORT-EHEALTH checklist (V 1.6.1).

## Abbreviations

MBAT: Mindfulness-Based Addiction Treatment
SES: socioeconomic status
